# Regularity of colour statistics in explaining colour composition preferences in art paintings

**DOI:** 10.1038/s41598-022-18847-9

**Published:** 2022-08-26

**Authors:** Shigeki Nakauchi, Hideki Tamura

**Affiliations:** Department of Computer Science and Engineering, Toyohashi University of Technology, 1-1 Hibarigaoka Tempaku, Toyohashi, Aichi 441-8580 Japan

**Keywords:** Psychology, Human behaviour

## Abstract

This study explores the role of colour statistics in painting preferences and tests the ‘matching-to-nature’ hypothesis which posits that the preference for the colour composition of paintings depends on the extent to which the paintings resemble the colour statistics of natural scenes. A preference judgement experiment was conducted with 31,353 participants using original and hue-rotated versions of 1,200 paintings. Multiple regression analyses were performed between the measured preferences and paintings’ colour statistics to reveal which colour statistics explained the preference data and to what extent. The colour statistics of art paintings that explained the preference data were compared to the colour statistics of natural scenes. The results identified the colour statistics that significantly contributed to explaining painting preferences, and the distributions of the paintings’ colour statistics systematically differed from those of natural scenes. These findings suggest that the human visual system encodes colour statistics to make aesthetic judgements based on the artistic merit of colour compositions, and not on their similarity to natural scenes.

## Introduction

Colour is among the most influential visual features in individuals’ preferences^1–3^. Art paintings are considered to purely reflect the artist’s taste or preference for colours. Essentially, the work is an expression of the artist’s personal aesthetic experience, and diversity is naturally expected, although there are non-personal trends for various styles and articulations that are influenced by social or environmental factors^4,5^. Despite the highly personal nature of art paintings, often linked to the notion that ‘*beauty is in the eyes of the beholder*’, there exist art paintings in which different individuals find similar aesthetic pleasure. Accordingly, some findings have suggested universality among art paintings^6^. There have been several attempts to show similarity in statistical properties between paintings and natural scenes as a basis of the universality, mainly in the spatial domain, for example, fractal-like scale invariant statistical properties^7–10^. The similarities between paintings and natural scenes led to the hypothesis linked to ‘*matching-to-nature*’, such that the painters reproduce the statistical regularities of natural scenes in their art paintings, to which the visual system is efficiently adapted^11–14^, to induce a kind of ‘aesthetic resonance’ between their paintings and viewers^6^.

Despite the limited analysis of the colour statistics of paintings, some important results have been presented. Notably, it has been argued that the distribution of colours in paintings, irrespective of their genre, resembles natural scenes. For example, three-dimensional volume in a colourimetric space formed by colours of pixels in paintings of various painters and typical natural scenes share a general tendency of an elongated shape in the blue-yellow direction, suggesting that both paintings and natural scenes as a whole tend to vary more in bluish–yellowish than in reddish–greenish contrasts^12,15,16^.

If, as previous studies^6–10^ argued, the aesthetic preference for art paintings is mediated by similarity to natural scenes, and if colour composition sufficiently influences the paintings' aesthetic preference, the matching-to-nature hypothesis might also arise for colour composition, that is, the preference for the colour composition of paintings depends on the extent to which the paintings resemble the colour statistics of natural scenes. Although colour preference has been mainly studied in the context of a single colour or a limited number of colours^1–3,17^, more recent studies have focused on the preference of colour composition in paintings^18–21^. These studies manipulated only colour composition by rotating the three-dimensional colour volume of unfamiliar paintings. Observers were asked to adjust the hue angle to obtain the best subjective visual impression^18^, to select their preference among pairs of hue-rotated versions of the same painting^19,21^, or to rate their personal evaluation of each original or hue-rotated painting presented^20^. Despite the minor differences in methodology, these studies commonly found that observers typically prefer a chromatic composition very close to the original, rather than one that is hue-rotated, even in the case of non-figurative or spatially scrambled paintings^19^, or patchwork images comprising different art paintings^21^. These results, showing the superiority of the colour composition of original paintings, may lead the hypothetical explanations that the superiority of original paintings could be attributed to colour statistics similar to those of natural scenes, and that the similarity may have been broken by the hue-rotation.

Two critical questions arise here. The first concerns the superiority of the original colour composition of a painting and the particular factors of this composition that determine observers’ preferences. One possible explanation may be based on the naturalness of the colour composition. A recent study^22^ tested the hypothesis that individuals have an aesthetic preference for chromatic compositions that have a degree of naturalness and found a close relationship between the perceived degree of naturalness and the degree to which participants preferred an image. Another possible factor could be the perceived richness of colour (how many colours participants perceived). However, no evident correlation has been found between preference and colour richness; people do not simply prefer colourful paintings^20^. A recent study^23^ focusing on the colour statistics of landscape paintings and photographs found significant correlations between colour statistics and visual aesthetics responses, although the authors tested only a limited number of images (15 paintings and 15 photographs). Attempts focusing on features other than colour have also been made to predict preferences by incorporating high-level features, such as a feature relating to artistic style (e.g., ‘abstract’ or ‘concrete’, ‘dynamic’ or ‘still’)^24,25^, or the preference data for the objects depicted within paintings^17,26^. Although the naturalness and richness of colour compositions, or other high-level features may contribute to individuals’ aesthetic judgements of paintings, the extent to which each of these factors explains the preferences remains unconfirmed. The second question relates to the universality among paintings and their similarity to natural scenes. As mentioned, paintings and natural scenes have been argued to share some common colour features^15,16^; however, it remains unclear to what extent paintings and natural scenes are similar, and how and to what extent these common features influence individuals’ preferences for paintings.

**Figure 1:**
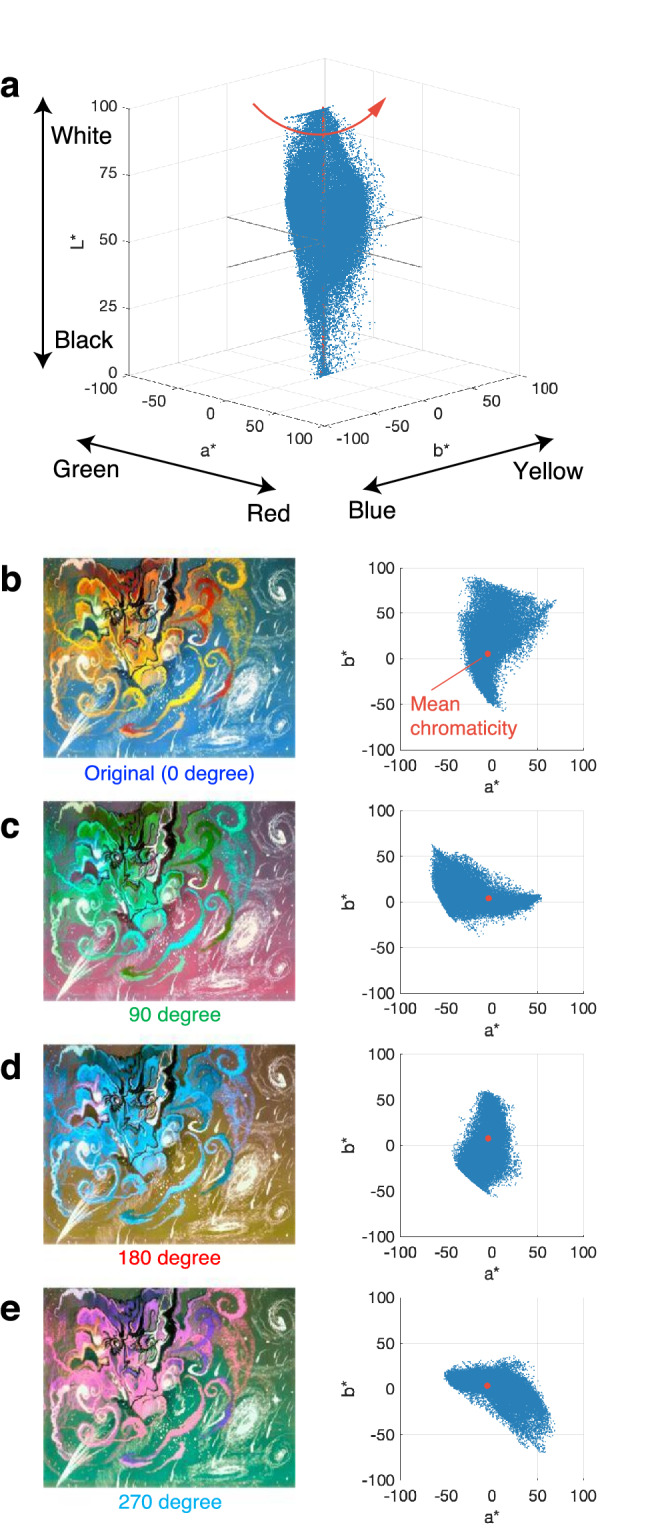
Manipulation of colour composition of art paintings by hue-rotation. (**a**) Colour gamut, a set of colour coordinates (L*, a*, b*) of pixels, displayed in the CIELAB colour space. Hue-rotated images were generated by rotating the colour gamut of the original counter clockwise around the centre of mass of the a*-b* plane of the gamut parallel to the L* axis. (**b**–**e**) Original (0°) and three of the hue-rotated images (90°, 180°, and 270°) and the colour gamut of them projected onto an a*-b* plane. Shown image is a typical example of an abstract painting by Stanisław Ignacy Witkiewicz (1885–1939) in 1918 (Source: WikiArt (https://www.wikiart.org/)).

This study aims to test the matching-to-nature hypothesis for the colour preference of art paintings. Specifically, we explored the role of colour statistics in preferences for paintings, focusing on the links among colour statistics, preferences for colour compositions, and the universality among paintings in terms of their similarity to natural scenes. We first conducted a preference judgement experiment with 31,353 participants that employed a four-alternative forced-choice (4AFC) paradigm by using the original (0°) and hue-rotated versions (90°, 180° and 270° in hue angle), as shown in Fig. 1, similar to a previous study^21^. Hue rotation was performed in the CIELAB colour space^27^, which represents colours by using the coordinates in a uniform colour space consisting of lightness variable L* and chromaticity indices a* and b* (Fig. 1a). L* indicates from black to white, negative a* corresponds with green, positive a* corresponds with red, negative b* corresponds with blue, and positive b* corresponds with yellow. Three of the hue-rotated images (Fig. 1c–e) were generated by rotating the colour gamut of the original (Fig. 1b), a set of points for each pixel coordinate shown by blue dots in Fig. 1a, by 90°, 180°, and 270° counter-clockwise around the centre of mass of the a*-b* plane of the image parallel to the L* axis.

**Figure 2:**
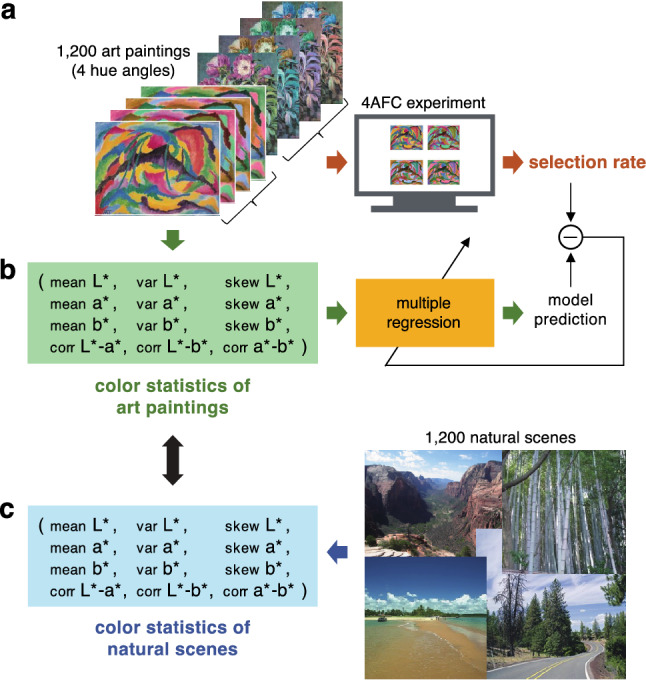
Analysis of colour statistics of art paintings and natural scenes to test the ‘matching-to-nature’ hypothesis on colour preference. (**a**) We collected the preference data (selection rate) for 1,200 sets of original and hue-rotated images of art paintings by 4AFC experiment. (**b**) The 1st to 3rd order of colour statistics of 1,200 paintings: mean, variance, and the skewness of colourimetric values in the CIELAB colour space: L*, a* and b*, and the correlation between two colourimetric values, L*–a*, L*–b*, and a*–b* were calculated. We then performed a multiple regression analysis on pairs of the measured selection rates and the colour statistics of each image to reveal which colour statistics explained the preference data and to what extent. (**c**) To test the ‘matching-to-nature’ hypothesis that the similarity between paintings and nature scenes is the basis of the superiority of original paintings, colour statistics of original paintings and natural images were compared.

First, we collected selection rates of the original and three hue-rotated images for 1,200 paintings collected from an internet image database^28^ (Fig. 2a). We also calculated the 1st to 3rd order of colour statistics of the 1,200 paintings: mean, variance, and the skewness of colourimetric values in the CIELAB colour space: L*, a*, and b*, and the correlation between two colourimetric values, L*–a*, L*–b*, and a*–b* (Supplementary information S1. Definition of the 1st–3rd order colour statistics). A recent study^23^ has attempted to show the relationship between simple image statistics including colour statistics and aesthetic experiences. However, as already pointed out, only a limited number of paintings and photographs were analyzed. We aim to explore the link between colour statistics and preference with a large number of paintings and observers.

We then performed a multiple regression analysis on pairs of the measured selection rates and the colour statistics of each image to reveal which colour statistics explained the preference data and to what extent (Fig. 2b). Next, to test the matching-to-nature hypothesis that the similarity between paintings and nature scenes is the basis of the superiority of paintings, we compared the colour statistics of original paintings with those of natural scenes. Specifically, we investigated the relationship between the contribution of the colour statistics to explain the preferences and their similarities to those of natural scenes (Fig. 2c).

## Results

### Robustness of superiority in preferences for the original colour composition of paintings

**Figure 3:**
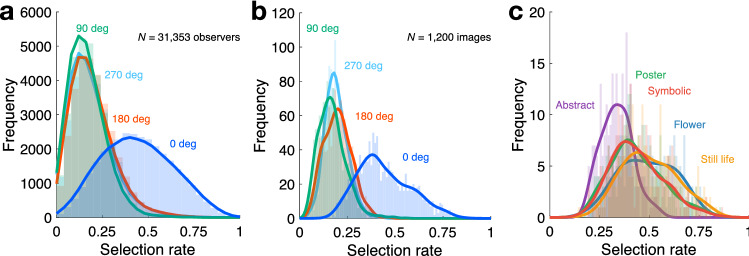
Distribution of the selection rates for the original and three hue-rotated images. (**a**) The selection rates of each hue angle across observers (*N* = 31,353). The horizontal axis indicates the average selection rate, and the vertical axis indicates frequency. The number of counts in each bin was determined from a 25-binned histogram. Lines are determined by fitting the kernel to the frequency histograms. (**b**) The selection rates of each hue angle across images (*N* = 1,200). The number of counts in each bin was determined from a 100-binned histogram. (**c**) The distributions of the selection rates for the original paintings (0°) displayed separately by genre.

Figure 3a shows the distribution of the average selection rates of each hue angle across observers (*N* = 31,353) collected in the 4AFC experiment. The average selection rate differed depending on the hue angles, Friedman’s test: χ^2^(3) = 24,095.7, *P* < 0.001. The average selection rate for the original images (0°) was significantly higher than that of the other hue angles (0° vs. 90°, *P* < 0.001; 0° vs. 180°, *P* < 0.001; 0° vs. 270°, *P* < 0.001; Wilcoxon signed-rank test with Bonferroni correction). The selection rate for the original images was above chance level, χ^2^(1) = 10,314.4, *P* < 0.001, confirming the superiority of original colour compositions observed in previous studies^18–21^.

The present study focused on the dependency of the selection rates on the paintings. Figure 3b shows the distribution of the selection rates across the images (*N* = 1,200), displayed separately for the original version and the three hue-rotated images. The average of the selection rates for the original images was significantly higher than for the other versions, χ^2^(3) = 1686.7, *P* < 0.001, Friedman’s test with Wilcoxon signed-rank test with Bonferroni correction.

Figure 3c shows the distribution of the selection rates for the original versions, displayed separately by genre (*abstract*, *poster*, *symbolic*, *flower,* and *still life*). A significant difference was noted between the average selection rates, with a large effect size among genres, Welch’s ANOVA: *F*(4, 586.2) = 104.6, *P* < 0.001, *η*_*p*_^2^ = 0.42, *ω*^2^ = 0.41. A comparison by genre revealed that the average selection rates were ordered as: *abstract* < *poster* = *symbolic* < *flowers* = *still life* (pairwise comparisons using Games-Howell test). Importantly, the average rate of the original abstract paintings was significantly above chance level (χ^2^(1) = 123.3, *P* < 0.001), despite the absence of object-related colour clues.

### Colour statistics of paintings and the effect of hue rotation

We analysed the colour statistics of the paintings used as stimuli in the experiment. The standard RGB (sRGB) coordinates of each pixel of 4,800 images (1,200 paintings × 4 hue angles) were transformed to the corresponding colourimetric values (L*, a*, and b* coordinates in the CIELAB colour space). Here, we focused on the 1st, 2nd, and 3rd order statistics of each colour coordinate and the correlation between pairs of two coordinates for the original version and the three hue-rotated images.

**Figure 4:**
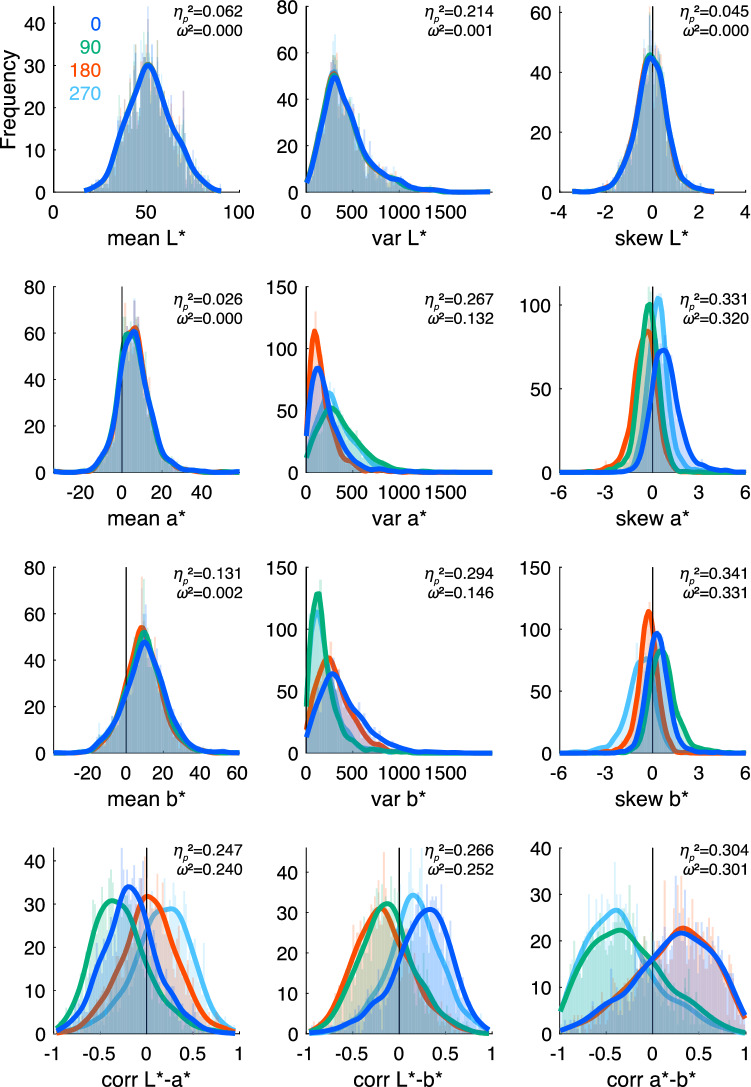
Distributions of the 12 types of colour statistics of art paintings (*N* = 1,200) displayed for each hue rotation angle. Originals are drawn by blue lines, 90° by green, 180° by red, and 270° by cyan. The magnitude of the difference among distributions is indicated by the effect size *η*_*p*_^2^ and *ω*^2^ displayed in each panel.

Figure 4 presents the distributions of the 12 types of colour statistics of the 4 hue angles × 1200 images, displayed separately for each hue angle (0°, 90°, 180°, and 270°). The extent to which the hue rotation affected the colour statistics (the magnitude of difference among the colour statistic distributions of different hue angles) was measured by the effect size of partial eta-squared (*η*_*p*_^2^) and omega-squared (*ω*^2^) and displayed in each panel. Manipulating the hue angle did not alter the L* coordinate (mean, variance, and skewness of L*) and mean of a* and b* in principle. Although there were small differences among the hue angles for these colour statistics due to the gamut-mapping process (in which the colours of the gamut were projected onto the closest displayable colours in the CIELAB colour space), the effect size according to hue rotation was negligible (e.g., *ω*^2^ < 0.01).

When the hue angle was rotated 90° or 270°, the a* and b* coordinates were interchanged. When rotated to 180°, the sign of each coordinate was reversed. Differences in the colour statistic distributions between a* and b* were reflected in changes due to the hue rotation of 90° and 270°, and the distributional asymmetry in each colour coordinate was reflected in changes due to the hue rotation of 180°. Therefore, the fact that the hue rotation mainly altered the variance and skewness of a* and b*, as well as the correlations, is a clear indication of the nature of the colour statistics of paintings. For example, due to the blue-yellow elongation of the colour gamut of art paintings^15,16^, the variance of b* tends to be larger than that of a* in the original versions. Another example is that the correlation between a* and b* shows positive bias corresponding to the observation that the colour gamut of paintings tends to be tilted clockwise^16^. These tendencies in the original art paintings were altered by hue rotation. In other words, if the colours used in paintings are distributed symmetrically around mean chromaticity, the hue rotation does not affect any of their colour statistics.

Notably, general trends in the colour statistics distributions of original paintings, for instance, difference in variance between a* and b*, or positive bias in skewness of a* and in the correlation between a* and b* and L* and b*, were observed regardless of genre (further details in Supplementary information S2. Typical examples of hue rotation in each genre, and S3. Distributions of the colour statistics of art paintings in each genre).

### Multiple regression analysis of colour statistics linking to the human preference data

The selection rates shown in Fig. 3b and the colour statistics shown in Fig. 4 vary with the paintings and with the rotated hue angle. To explore this relationship, a multiple regression analysis was conducted for pairs of the colour statistics of the 4,800 images as explanatory variables and the selection rates for those images as target variables.

**Figure 5:**
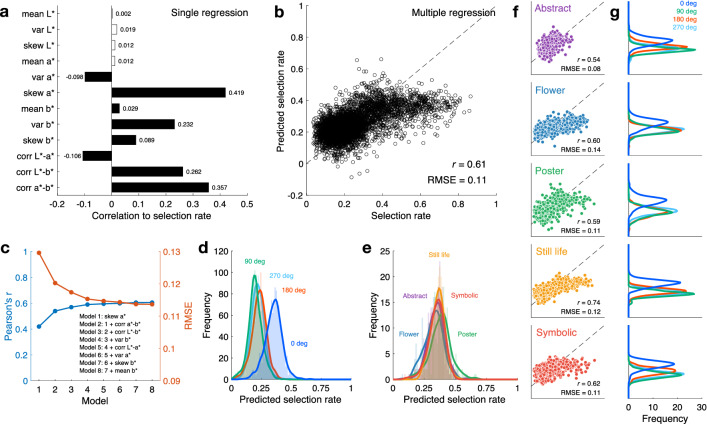
Multiple regression analysis of the colour statistics to explain the selection rates. (**a**) Correlation coefficients (Pearson’s *r*) between the selection rates and 12 types of colour statistics determined by a single regression analysis. The filled bars indicate statistically significant independent variables. (**b**) Scatter plot of the human selection rates and the selection rates predicted by the multiple regression model with the eight selected colour statistics as the independent variables. Each circle represents one image. (**c**) Correlation coefficients (blue line) and RMSE (red line) as a measure of the model performance when the number of independent variables was altered. (**d**) The distribution of the model outputs as of predicted selection rates in each hue angle illustrated in the same manner as Fig. 3b. (**e**) Distributions of the predicted selection rate for the original images displayed separately for each genre illustrated in the same manner as Fig. 3c. (**f**) The scatter plots of the human selection data and the model outputs are separately displayed for each genre. (**g**) Distributions of the selection rates predicted by the model in each hue angle for each genre.

First, correlation coefficients with individual colour statistics were determined by a single regression analysis. Eight among the 12 types of colour statistics showed a significant correlation with the selection rate (Fig. 5a; illustrated by the filled bars). Among them, some of the colour statistics reflecting the nature of the colour gamut of paintings significantly contributed to explain the human preference data, for example, the skewness of a* (Pearson’s *r* = 0.419, *P* < 0.001) and the correlation between a* and b* (Pearson’s *r* = 0.357, *P* < 0.001) and L* and b* (Pearson’s *r* = 0.262, *P* < 0.001).

Next, we constructed a multiple regression model to explain the selection rates of 4,800 images using all 12 types of colour statistics. To find meaningful colour statistics as independent variables, variables were selected using a backward elimination method^29^. The selected independent variables included eight types of colour statistics: mean of b*, variance of a* and b*, skewness of a* and b*, and correlations between L* and a*, L* and b*, and correlation between a* and b*. These selected colour statistics were identical to those showing a significant correlation in the single regression analysis depicted in Fig. 5a. To show the accuracy of the multiple regression model, Fig. 5b presents a scatter plot of the selection rates measured by the experiment from the observers and those predicted by the model with the eight selected colour statistics. The obtained multi-regression model showed a significant correlation with the human preference data (Pearson’s *r* = 0.61, *P* < 0.001). The model did not show multicollinearity among the colour statistics since all variance inflation factors (VIF^30^) were less than 10. Moreover, when the variables were selected using the forward selection method^29^ or stepwise method^29^, the same model with eight variables was obtained as in the backward elimination method, and this model had the lowest Akaike information criterion (AIC). Figure 5c shows the dependency of the model performance on the number of independent variables when the number of variables was altered by the forward selection method^29^. The model performance measured by the correlation between the model outputs and the selection rates tended to be saturated when the number of variables reached three or more, again implying the relevance of the skewness of a* and the correlation between a* and b* and L* and b* to explain the human preference data.

Figure 5d shows the distributions of the selection rates predicted by the model for the original and three hue-rotated images, corresponding to the human data presented in Fig. 3b. The greater preference for original colour composition (i.e., highest selection rates for the original versus hue-rotated versions) was reproduced only from the colour statistics of the images (χ^2^(3) = 1517.0, *P* < 0.001, Friedman’s test with Wilcoxon signed-rank test using Bonferroni correction). Figure 5e demonstrates the distributions of the model outputs for the original images separately by genre, similar to Fig. 3c. The dependency of the selection rates on the genres was not reproduced in the model outputs (*F*(4, 594.4) = 16.2, *P* < 0.001, *η*_*p*_^2^ = 0.10, *ω*^2^ = 0.09, Welch’s ANOVA).

Figure 5f,g show the results separately replotted for each painting genre. For all genres, the model outputs and selection rates were significantly correlated (*P* < 0.001), and the original superiority of the preferences was reproduced in the model outputs (*abstract*: χ^2^(1) = 173.4; *flowers*: χ^2^(1) = 104.0; *posters*: χ^2^(1) = 176.8; *still life*: χ^2^(1) = 205.4, *symbolic*: χ^2^(1) = 173.4). When model performance was evaluated by root mean square error (RMSE), among all five genres, the selection rate for the *abstract* paintings was most explained (RMSE = 0.08), while the selection of the paintings in the *flowers* genre was least explained (RMSE = 0.14).

### Similarities between the colour statistics of paintings and natural scenes

**Figure 6:**
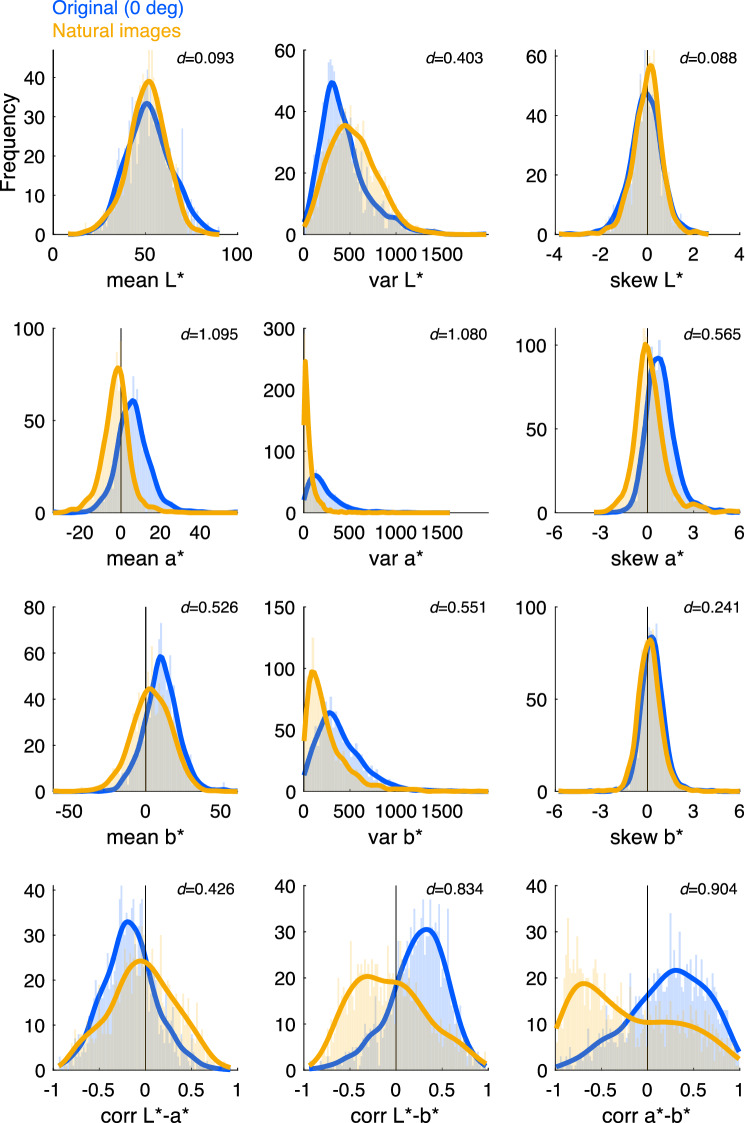
Distribution of colour statistics for the original art paintings and natural scenes. Blue and yellow lines show paintings and natural scenes, respectively. The data for natural scenes were collected from an internet image database (SUN database). A total of 1,200 scenes categorised as ‘outdoor natural’ were randomly selected. The effect size *d* as a measure of group difference was determined by t-test for each pair of distributions and is shown in each panel.

A multiple regression analysis of the effect of the colour statistics on the selection rates revealed that some colour statistics play a fundamental role in explaining preferences for the colour composition of paintings. What are the mechanisms behind the influence of these colour statistics on such preferences? One plausible hypothesis is the similarity of the colour composition in both natural scenes and paintings^11–13^. To test this matching-to-nature hypothesis, the distributions of the colour statistics were directly compared between the original paintings used as stimuli in the experiment and natural scenes collected from an internet image database^31^. A total of 1,200 scenes categorised as ‘*outdoor natural*’ were randomly selected, and the 12 types of colour statistics for these scenes were calculated. Figure 6 illustrates the distributions of the 12 types of colour statistics for the paintings (blue) and natural scenes (yellow), with the effect size (Cohen’s *d*) determined by a t-test. Previous studies^15,16^ have argued that the colour gamut of both paintings and natural scenes are elongated. This tendency was indeed observed in the distribution of the variance of a* and b*, that is, the mean of variance of b* was larger than that of a* for both the paintings and natural scenes. Further, the colour gamut in the CIELAB a*–b* plane for paintings and natural scenes tends to be tilted but in different directions: clockwise for paintings and anticlockwise for natural scenes. This was also duplicated in the distribution of the correlation between a* and b*, that is, the correlation tended to be positive for paintings and negative for natural scenes^16^. However, except for some colour statistics (e.g., mean of L* and skewness of L* and b*), significant differences were observed between the distributions of the paintings and natural scenes.

To confirm the consistency of the results described above, we analysed the colour statistics distributions for another 1,200 natural scenes collected from a different database^32^ (further details in Supplementary information S4. Consistency of the results confirmed by another natural images database). The image set contains natural scenes without man-made objects or people, which were expected to be similar to the images we previously analysed. The findings mentioned above were replicated with the different natural scenes set; the distributions of relevant colour statistics explaining preferences, such as the skewness of a* or correlation between a* and b*, correlation between L* and b*, or variance of b*, showed a systematic difference from those of natural scenes. Further, general trends of the dissimilarities in colour statistics distributions between the natural scenes and paintings were replicated in a different set of natural scenes.

Table 1 summarises the analysis results of the 12 types of colour statistics of paintings we focused on, including the absolute value of the standard partial regression coefficients of the model as a degree of contribution to explain the human preference data obtained from the multiple regression analysis, the effect size of the hue rotation (*η*_*p*_^2^ and *ω*^2^), and the magnitude of dissimilarity between the colour statistics of art paintings and those of natural scenes (Cohen’s *d*). To illustrate the degree to which the importance of the partial regression coefficients of the model is linked to these factors, we examined the correlation coefficients between the coefficients of the model and the amount of effect by hue rotation, and between the coefficients and the dissimilarity to natural scenes. Consequently, correlation between the degree of contribution to explain the human preference data of the model and the effect size of hue rotation was significant (Pearson’s *r* = 0.715, *P* = 0.009 for *η*_*p*_^2^; Pearson’s *r* = 0.795, *P* = 0.002 for *ω*^2^), while not significant for dissimilarity to natural scenes (Pearson’s *r* = 0.398, *P* = 0.200 for Cohen’s *d*).

**Table 1:** Summary of the results of the analysis of colour statistics.

Color stats.	Contribution to preference	Effect of hue rotation (Fig. 4)		Dissimilarity to natural scene (Fig. 6)
	Partial regression coefficient (absolute value)	Effect size *η*_*p*_^2^	Effect size *ω*^2^	Effect size d
skew a*	0.049	0.331	0.320	0.565
corr a*–b*	0.046	0.304	0.301	0.904
corr L*–b*	0.026	0.266	0.252	0.834
var b*	0.023	0.294	0.146	0.551
skew b*	0.013	0.341	0.331	0.241
var a*	0.010	0.267	0.132	1.080
corr L*-a*	0.009	0.247	0.240	0.426
mean b*	0.003	0.131	0.002	0.526
var L*	–	0.214	0.001	0.403
mean L*	–	0.062	0.000	0.093
skew L*	–	0.045	0.000	0.088
mean a*	–	0.026	0.000	1.095

## Discussion

This study showed that some relevant colour statistics can explain human preferences for the colour composition of paintings. The skewness of a* and the correlation between a* and b* and L* and b* largely contributed to explaining the preference data. However, since the colour statistics did not contain the genre-dependent features, dependency of the selection rates on the genre could not be explained by the multiple regression model. The model performance, shown as a scatter plot in Fig. 5b, indicates a saturating trend, which is due to the model's failure to correctly predict the human data, especially when the measured selection rates were relatively high (> 60%). A similar saturating trend is observed in the predicted selection rates shown in Fig. 5e when compared to the measured selection rates shown in Fig. 3c. Specifically, as shown in Fig. 5f, the performance of the model was high (RMSE is small) for *abstract* paintings, where the effects of genre are small, and low (RMSE is large) for the *flowers* genre, where factors related to figurative elements have a large impact. This clearly shows the effects of the colours associated with familiar objects depicted in the paintings^17,26^. Paintings in the *flowers* and *still life* genres are generally realistic or figurative in their pictorial composition and depict familiar objects and the colours associated with them. Meanwhile, paintings in the *posters* and *symbolic* genres are not realistic but often contain familiar objects, such as faces or flowers, as compositional elements. If the objects depicted in paintings of these genres are transformed to contain unfamiliar or unnatural colours through hue rotation, it is highly likely that observers’ preferences will be influenced by these adjusted colours^22^. Yet, abstract paintings generally do not depict objects that can be specifically associated with colours, and it is highly unlikely that the colours associated with objects will provide clues for preference judgements. Therefore, this may be why the selection rate of the abstract paintings was lower than that of other genres. Importantly, the colour composition of the original abstract paintings was still selected more than the hue-rotated versions, and the average rate was significantly above chance level (χ^2^(1) = 123.3, *P* < 0.001), despite the absence of such object-related colour clues. This is exactly what this study aimed to clarify, and the question deliberated relates to what kind of colour cues result in a preference for the original colour composition. In other words, paintings can have common colour statistics regardless of their genre, and other factors such as the colours associated with specific objects may contribute to representing the genre.

The selection rate for the original and hue-rotated images was explained by the 1st to 3rd order colour statistics of these images more fully than we had expected, even though the colour statistics did not carry any information about the objects depicted or the spatial arrangement of the images. The multiple regression model was able to regress the selection rate with an accuracy of *r* = 0.61 using only eight different colour statistics after variable selection. The contributions of each of these eight colour statistics to explaining the selection rate differed, especially the skewness of a* and the correlations between a*-b* and L*-b*, which showed strong correlations with the selection rate.

Most importantly, our findings shared in Table 1 also show systematic differences between natural scenes and paintings in terms of how the relevant colour statistics are linked to participants’ preferences, suggesting that the preferred colour composition of paintings does not necessarily resemble that of natural scenes. The colour statistics that largely contributed to explaining the human preference data, such as the skewness of a* and the correlation between a*-b* and L*-b*, were in general affected by hue rotation, although not all colour statistics affected by hue rotation contributed to the same extent. For example, the skewness of b* was affected by hue rotation to the same extent as the skewness of a*, although their contributions to explaining the human preference data differed. The distributions of the relevant colour statistics explaining participants’ preferences did not necessarily resemble those of the natural scenes either. The distribution of the skewness of a* in the paintings was positively biased but was not so in the natural scenes, and the correlation between a* and b* showed a similar tendency, resulting in the larger dissimilarity of these colour statistics between paintings and natural scenes. These trends were replicated for a different set of natural scenes, suggesting that the dissimilarity of colour statistics between paintings and natural scenes is a universal characteristic (Supplementary information S4. Consistency of the results confirmed by another natural image database).

The correlation between a* and b* significantly explained the preference data. This colour statistic directly reflects the shape of the colour gamut of paintings. It was revealed that the colour gamut of different paintings exhibits a similar blue-yellow elongation and is tilted slightly clockwise^16^. The distribution of the colour statistics shown in Fig. 6 reveals that art paintings tend to have a positive correlation between a* and b*, which is consistent with previous reports of a slight clockwise tilt in the colour gamut. Similarly, the correlation between L* and b* of paintings tends to be positive, which implies that the gamut of paintings tilts towards the yellow direction for brighter colours and towards the blue direction for darker colours. These distortions in the shape of the gamut may be related to the gamut of the colour materials^33^ or other cognitive biases. It is important to emphasise that neither tendency is found in natural images and that the gamut of a painting does not necessarily resemble that of a natural image.

One recent study found a close relationship between the perceived degree of naturalness and the degree to which people favour an image^22^, while our results have shown that the preferred paintings do not resemble the natural scenes in terms of colour distribution. This may seem contradictory, but it also means that perceiving naturalness in a colour composition does not necessarily imply that the colour composition resembles a natural scene. What we speculate here is that perceiving naturalness in paintings is dominated by its archetypal nature as the colour composition of paintings, which partially resembles natural scenes but is largely regulated by the painting’s own colour composition.

Traditionally, colour preferences have been examined in the context of single colours^1–3^, while our results imply that preferences for colour compositions can be explained by the combination of colour statistics, not only by mean hue. Particularly, the 3rd order statistic, skewness, is an important indicator. How does the skewness of visual features relate to perception? Reportedly, when the preference for different images with manipulated luminance skewness was examined, images with positive skewness, which is more common in natural images^34^, were not preferred, but images close to zero were^35^. Our findings showed that the skewness of a* significantly contributed to the selection preferences from the multiple regression analysis, and simultaneously, there was a clear difference between the distributions of the natural images and paintings. These results clearly differ from what the matching-to-nature hypothesis predicts.

What, then, are the perceptual implications of the skewness of a*, that is, the asymmetry of the distribution of the red–green colour opponent responses? The positive skewness of a* means that there are a few highly saturated red colours in paintings, and there indeed exists a bias towards redder colours in paintings or other artworks^16,36^. The psychological meaning of the skewness of colour statistics is unclear^23^. Could it be related to the biological origins of aesthetic experiences or preferences? Humans have been using red pigments for body painting by 164,000 years ago^37^, and it is thought that they were used for symbolic communication, such as in rituals, rather than for any specific practical reason^38^. The visual saliency of red has been considered an important reason for its choice, in addition to environmental factors related to the pigment material^38^. This suggests that red has a special meaning for human beings in terms of transmission of information to others, rather than cognitive factors such as the recall of concrete objects; this may have been linked to artistic representation, and in turn to preferences. However, the speciality of red, or specifically, the speciality of the skewness of a* and its perceptual effects, remain unclear. Although we have shown that, as a general trend, the skewness of a* differs between natural images and art paintings, certain natural scenes with a positive skewness of a*, as in paintings, may be associated with biological values.

There are several limitations to our approach. Our experimental design allowed us to measure the relative preference among four hue-rotated versions of individual paintings; however, it did not reveal absolute preferences for individual paintings. Studies on preferences for single colours have argued that a universal preference for blue hues exists^1–3^. Accordingly, the absolute colour preference for paintings should be affected by the mean hue, although our experiments were designed to eliminate these effects. Other than colour statistics, observers' familiarity with paintings would be another possible factor affecting preference. In our experiment, we did not collect data on familiarity. One who is familiar with a painting may likely choose their preferred image according to their memory of the painting’s original version. It remains to be clarified, therefore, to what extent the various colour statistics we identified, the basic colour statistics such as mean hue, and observers' familiarity with paintings, influence individuals’ preferences. The preference for paintings cannot be fully explained by colour statistics alone. For example, it has been reported that preferences for paintings are well-predicted by preferences for the objects depicted within them^26^. The question on the extent to which colour statistics, among the various factors involved, influences preferences for paintings also remains to be explored in future work. Nonetheless, we expect that our findings that colour statistics contribute to preferences for paintings, and that the colour statistics of paintings do not necessarily resemble those of natural scenes, are major contributions to the scientific understanding of preferences for paintings.

## Methods

### Observers

Overall, 31,353 Japanese observers participated in an online survey; among them, 30,777 provided their personal information (16,837 men and 13,940 women; aged 15–97, mean = 46.6 years, *SD* = 15.4) and 576 did not. The observers agreed to participate in the study before engaging in the experiment.

### Art paintings

We collected 1,200 images of art paintings from WikiArt^28^, including 240 images each from five different genres (*abstract*, *flowers*, *posters*, *symbolic*, and *still life*). The images were resized to 250 px on the long side, maintaining the aspect ratio of the originals. Each testing set comprised 24 randomly selected images (eight from three different genres). In total, 50 testing sets were prepared. For each stimulus in the testing set, the standard RGB (sRGB) coordinates of each of the original images were transformed to the corresponding colour coordinates (L*, a*, b*) in the CIELAB colour space^27^. Thus, we used 4,800 images as experimental stimuli including the originals and three hue-rotated versions.

### Natural scenes

The data for natural scenes were collected from a SUN database^31^, which comprised a collection of annotated images covering diverse environmental scenes, places, and objects. A total of 1,200 scenes categorized as ‘*outdoor natural*’ were randomly selected as comparison data. The images were resized, and their colour coordinates were calculated in the same way as for the painting data.

### Procedure and task

The observers were randomly assigned to one of the 50 testing sets in the online survey. On the survey screen, four images of the same painting with different hue rotation angles were presented in a 2 × 2 arrangement at four random locations. The observers were asked to select their preferred image from among the four (4AFC). No other instruction was given to the observers to influence their selections. After the observer responded, the next trial was conducted. The trial was repeated 24 times (eight images × three genres) with different stimuli in the testing set (one trial per image). The order of the 24 trials in the same testing set was identical among the observers, although it was randomly scrambled to avoid any bias. We obtained selection rate data for 4,800 images (1,200 paintings × 4 hue angle rotations) from 31,353 observers, with an average of 627 responses per stimulus (mean = 627, SD = 25, range: 569–678). The average selection rate for each hue angle was used as an index of preferences for the paintings. The distributions of the selection data were illustrated by histograms with the lines of the kernel density functions estimated by the ‘*ksdensity*’ function of MATLAB R2020b (version 4.0.4).

### Colour statistics of art paintings and natural scenes

To define the colour statistics of the paintings and natural scenes, the 1st to 3rd-order statistics (i.e. mean, variance, and skewness) were computed by each L*, a*, and b* coordinate (means of L*, a* and b*; variances of L*, a* and b*; skewness of L*, a* and b*), and correlation coefficients (Pearson’s *r*) were computed between every two combinations of the three coordinates (correlations between L* and a*, L* and b*, and a* and b*). The definition of the colour statistics is presented in Supplementary information S1. The distributions of these 12 types of colour statistics of original and hue-rotated paintings are shown by histograms with the lines of the estimated kernel density functions (Fig. 4), and the distributions for the natural scenes are similarly illustrated (Fig. 6).

### Multiple regression analysis

We first performed a single regression on the selection rates according to each of the 12 types of colour statistics of 4,800 images, as shown in Fig. 5a. We then performed multiple regression analysis on the selection rates according to these 12 types using backward data entry^29^. As indices of the prediction accuracy, Pearson’s *r* and the RMSE were computed for the overall data (Fig. 5b), for various numbers of independent variables (Fig. 5c), and for each genre (Fig. 5f).

### Statistical analysis

The averages of the selection rates for each image category (original and 90°, 180°, and 270° hue-rotated) were analysed with a multinomial test and Friedman’s test, and multiple comparisons were performed with the Wilcoxon signed-rank test using Bonferroni correction. The average of the selection rates for each painting genre (*abstract, flowers, posters, still life,* and *symbolic*) was analysed with a multinomial test and one-way analysis of variance (Welch’s ANOVA), and multiple comparisons were performed with the Games-Howell test. Twelve types of painting colour statistics were analysed by one-way ANOVA with the hue-rotation angle as a factor and by *t*-test for image type (paintings or natural scenes) as a factor to determine the effect size (partial *η*^2^, *ω*^2^ and Cohen’s *d*) in terms of the magnitude of differences among the groups, since *p-*values are often meaningless for a large sample^39,40^. All the statistical analyses were performed using MATLAB R2020b (version 4.0.4) and the statistical software JASP (version 0.16.1)^41^.

### Ethical approval and informed consent

All experimental procedures were in accordance with the ethical principles outlined in the Declaration of Helsinki and approved by the Committee for Human Research at the Toyohashi University of Technology. The experiment was strictly conducted in accordance with the approved guidelines of the committee. Informed written consent was obtained from participants after procedural details were explained.

## Supplementary Information

Supplementary Information.

## Data Availability

All datasets generated during this study are included within this article. The analysed data are available in full from the corresponding author upon reasonable request.
