# Supplementary material for: Regularity of colour statistics in explaining colour composition preferences in art paintings
Source: Sci Rep. 2022 Aug 26;12:14585. doi: 10.1038/s41598-022-18847-9 (PMC9418166; doi:10.1038/s41598-022-18847-9)
Supplement: Supplementary file 1 — Supplementary Information. [file 41598_2022_18847_MOESM1_ESM.pdf]

Supplementary information

Regularity of colour statistics in explaining colour composition preferences in art paintings

Shigeki Nakauchi[1*] and Hideki Tamura[1]

[1]Department of Computer Science and Engineering, Toyohashi University of Technology, Toyohashi 441-8580, Japan

***Corresponding author**
Email: nakauchi@tut.jp

S1. Definition of the 1st – 3rd order colour statistics

Colour of each pixel of a given image at position $(1 \leq i \leq W, 1 \leq j \leq H)$ is described as a triplet of colour coordinates in the CIELAB colour space as $\{L^*(i,j), a^*(i,j), b^*(i,j)\}$, where W is the width and H is the height of the image in pixel number. Definition of the 1st, 2nd, and 3rd order statistics for the colour distribution of the image is given as follows:

1st order statistics (mean)

$$mean\ L^* = \frac{1}{WH}\sum_{i=1}^{W}\sum_{j=1}^{H} L^*(i,j),$$

$$mean\ a^* = \frac{1}{WH}\sum_{i=1}^{W}\sum_{j=1}^{H} a^*(i,j),$$

$$mean\ b^* = \frac{1}{WH}\sum_{i=1}^{W}\sum_{j=1}^{H} b^*(i,j).$$

2nd order statistics (variance)

$$var\ L^* = \frac{1}{WH}\sum_{i=1}^{W}\sum_{j=1}^{H} \{L^*(i,j) - mean\ L^*\}^2,$$

$$var\ a^* = \frac{1}{WH}\sum_{i=1}^{W}\sum_{j=1}^{H} \{a^*(i,j) - mean\ a^*\}^2,$$

$$var\ b^* = \frac{1}{WH}\sum_{i=1}^{W}\sum_{j=1}^{H} \{b^*(i,j) - mean\ b^*\}^2.$$

2nd order statistics (correlation)

$$corr\ L^*{-}a^* = \frac{1}{WH}\frac{1}{\sqrt{var\ L^* \times var\ a^*}}\sum_{i=1}^{W}\sum_{j=1}^{H} (L^*(i,j) - mean\ L^*)(a^*(i,j) - mean\ a^*),$$

$$corr\ L^*{-}b^* = \frac{1}{WH}\frac{1}{\sqrt{var\ L^* \times var\ b^*}}\sum_{i=1}^{W}\sum_{j=1}^{H} (L^*(i,j) - mean\ L^*)(b^*(i,j) - mean\ b^*),$$

$$corr\ a^*{-}\ b^* = \frac{1}{WH}\frac{1}{\sqrt{var\ a^* \times var\ b^*}}\sum_{i=1}^{W}\sum_{j=1}^{H} (a^*(i,j) - mean\ a^*)(b^*(i,j) - mean\ b^*).$$

3rd order statistics (skewness)

$$skew\ L^* = \frac{1}{WH}\frac{1}{\left\{\sqrt{var\ L^*}\right\}^3}\sum_{i=1}^{W}\sum_{j=1}^{H}\{L^*(i,j) - mean\ L^*\}^3\ ,$$

$$skew\ a^* = \frac{1}{WH}\frac{1}{\left\{\sqrt{var\ a^*}\right\}^3}\sum_{i=1}^{W}\sum_{j=1}^{H}\{a^*(i,j) - mean\ a^*\}^3\ ,$$

$$skew\ b^* = \frac{1}{WH}\frac{1}{\left\{\sqrt{var\ b^*}\right\}^3}\sum_{i=1}^{W}\sum_{j=1}^{H}\{b^*(i,j) - mean\ b^*\}^3\ .$$

S2. Typical examples of hue rotation in each genre

To illustrate the effects of hue rotation on each painting genre, supplementary Fig.S1 shows the typical examples of original and three hue-rotated versions (90°, 180°, and 270° in hue angle).

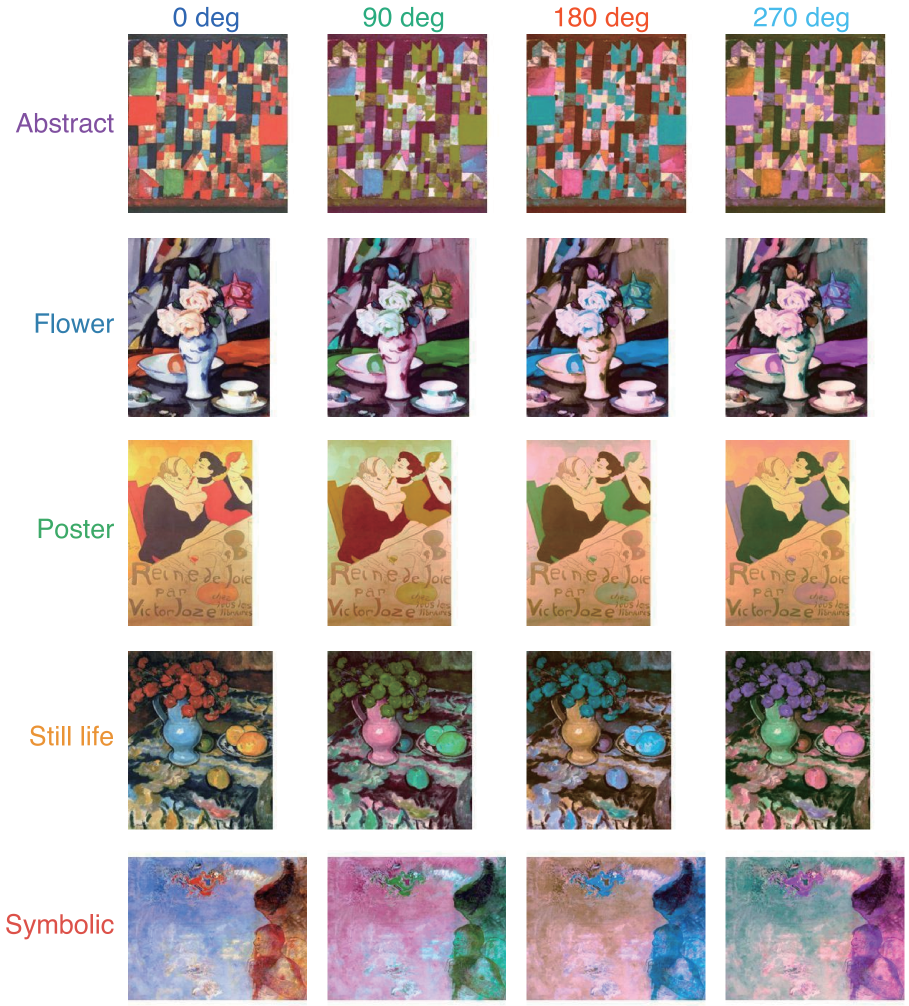

Supplementary Fig.S1 Typical examples of original and three hue-rotated versions of each genre. Abstract: Paul Klee (1903-1940) in 1921; Flower: Samuel Peploe (1871-1935) in 1923; Poster: Henri de Toulouse-Lautrec (1864-1901) in 1892; Still life: Józef Pankiewicz (1866-1940) in 1909-1910; Symbolic: Odilon Redon (1840-1916) in 1905. Source: WikiArt (https://www.wikiart.org/) .

S3. Distributions of the colour statistics of art paintings in each genre

We visualized the colour statistics distribution for each of the five genres of original paintings in Supplementary Fig.S2. Effect size of the genre seems to be relatively small (e.g., $\omega^2 < 0.05$) in general. The tilt of the colour gamut reflected in the correlation between *a** and *b** shows similar trends regardless of genre. Skewness of *a** shows a medium level effect size of the genre; however the general trend in the positive bias is observed in all genres. Some types of colour statistics, for example, variances of *L**, *a**, and *b** demonstrate dependency on the genre, which could be a factor in the different selection rate by genre shown in Figure 3c. However, the genre-independent trend of selectively preferring originals could be attributed to the small genre-dependent differences in the colour statistics, for example, the correlation of *a**-*b** or *L**-*b**, and skewness of *a**.

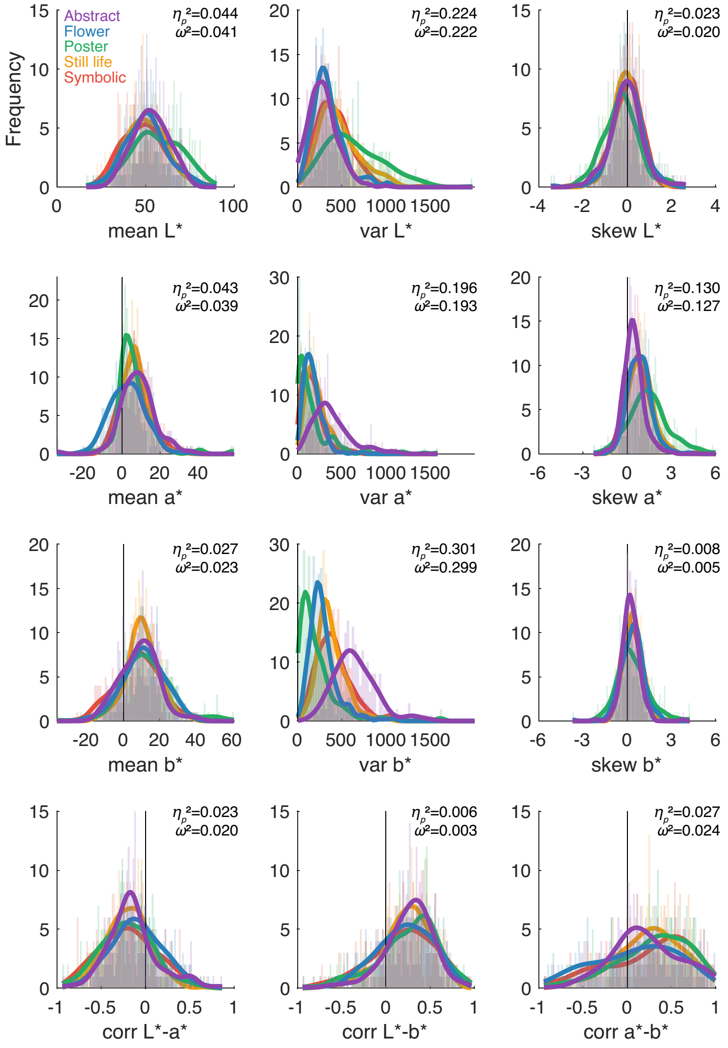

Supplementary Fig.S2 Distributions of the 12 types of colour statistics of original paintings displayed in each genre.

S4. Consistency of the results confirmed by another natural image database

To confirm the consistency of the results, we analysed the distributions of colour statistics for another 1,200 natural images data collected from the database at Center for Perceptual Systems, University of Texas at Austin (Geisler and Perry, 2011; https://natural-scenes.cps.utexas.edu/db.shtml). This database contains natural scenes without man-made objects or people, which were expected to be similar to the image sets categorized as '*outdoor natural*' in the SUN database shown in Figure 6. Furthermore, the database provides rich metadata (shutter speeds, F-number, etc.) and pixel sensitivity functions of colour sensors of the camera (Nikon D700 Digital SLR) which allow us to accurately convert the data to colorimetric values, for example, L^*, a^*, and b^*.

Supplementary Fig.S3 illustrates the distributions of colour statistics of art paintings which are identical to those in Figure 6 (blue lines) and natural scenes from Geisler and Perry's natural scenes database (red lines) that we newly analysed. Dissimilarity between paintings and natural scenes were measured by Cohen's d for each pair of colour statistics and shown in each panel in the same manner as Figure 6. Our argument was that the distributions of relevant colour statistics of art paintings explaining their preferences, such as the skewness of a^* or correlation between a^* and b^*, correlation between L^* and b^*, or variance of b^*, are different from those of natural images. This nature was duplicated in the newly analyzed natural scenes as shown in Fig.S3; for example, positive bias of skewness of a^* and the correlation between a^* and b^* as relevant features of statistical regularities in art paintings were not reproduced in the natural images. Instead, skewness of a^* is distributed around zero, and the a^*-b^* correlation shows negative-biased distribution in the natural scenes.

To demonstrate the consistency of the results more quantitatively, we compared the dissimilarities (Cohen's d) calculated for both databases, as illustrated by a scatter plot in Supplementary Fig.S4. The general trend, not only the specific colour statistics, of the dissimilarities between the natural images and paintings is very similar and Cohen's d in these cases were significantly correlated ($r = 0.615$, $p = 0.033$). This fact, therefore, strongly supports the consistency of our conclusion about the dissimilarity between natural images and paintings.

Reference

Geisler, W. S. & Perry, J. S. Statistics for optimal point prediction in natural images. ***J Vision*** 11, 14–14 (2011). https://doi.org/10.1167/11.12.14

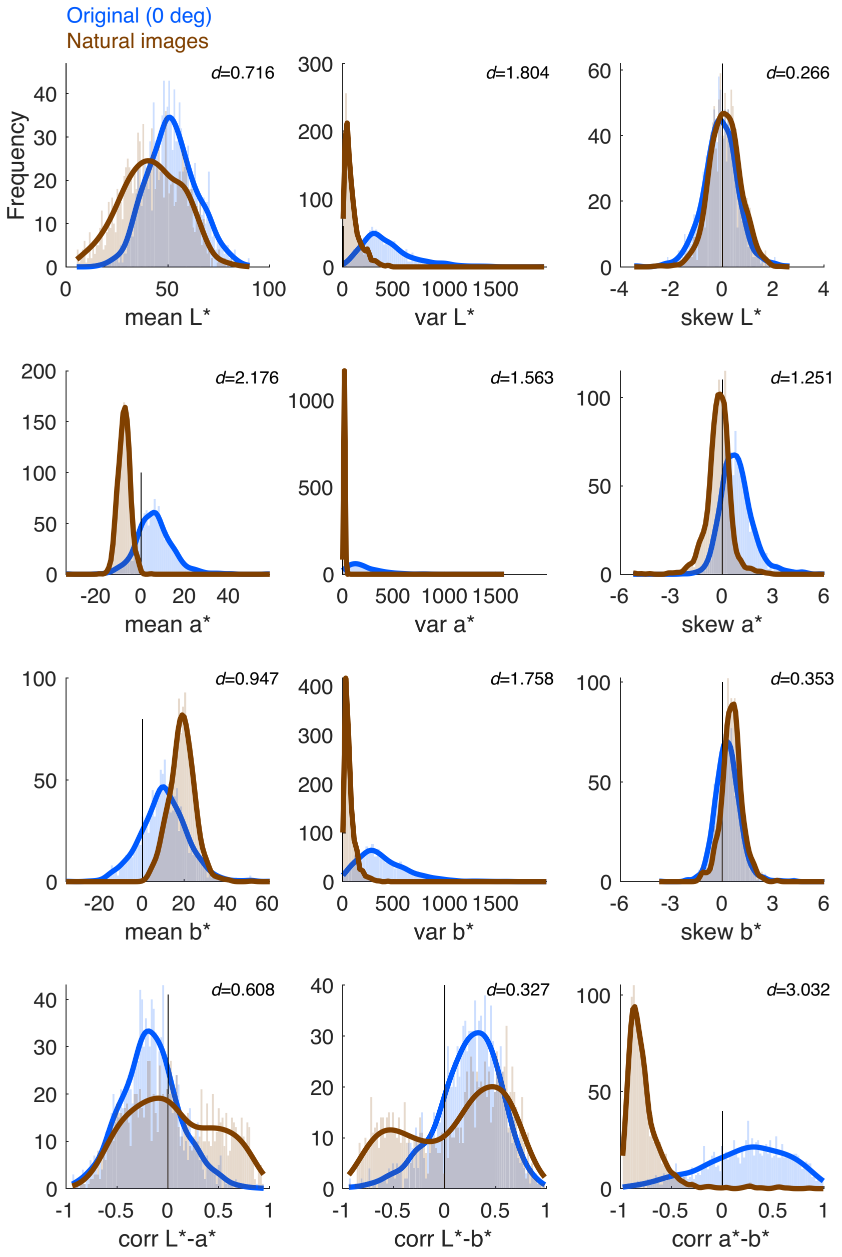

Supplementary Fig. S3. Comparison of colour statistics distributions: art paintings and natural scenes from Geisler and Perry's natural scene database.

Blue and red lines show paintings which are identical to those shown in Figure 6 and natural scene collected from Geisler and Perry's natural scenes database, respectively. The effect size Cohen's *d* as a measure of group difference was determined by t-test for each pair of distributions and is shown in each panel in the same manner as Figure 6.

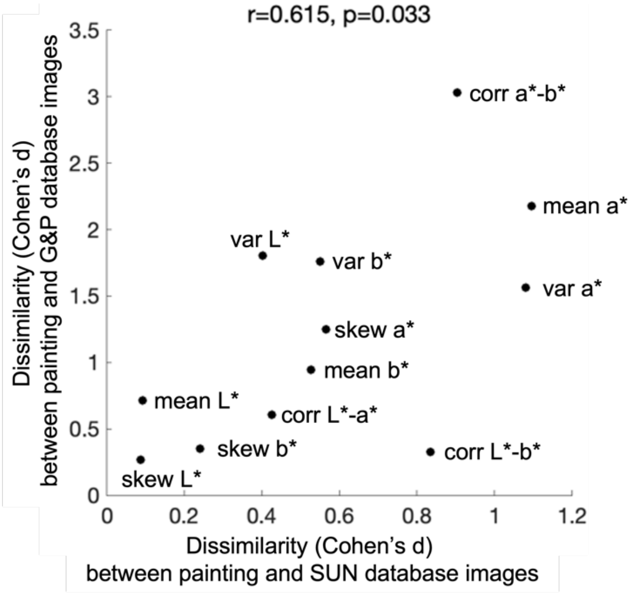

Supplementary Fig.S4. Scatter plot of dissimilarity to art paintings (Cohen' *d*) of natural images in the SUN database and Geisler and Perry's natural scene database
